# Lumiracoxib for acute postoperative dental pain: a systematic review of randomized clinical trials

**DOI:** 10.1590/S1516-31802011000500009

**Published:** 2011-09-01

**Authors:** Ricardo Carvalho Lopes Silva, Rachel Riera, Humberto Saconato

**Affiliations:** I MSc. Dental Surgeon. Former master's degree student at Universidade Federal de São Paulo (Unifesp), São Paulo, Brazil.; II MD, MSc. Attending Physician at Universidade Federal de São Paulo (Unifesp), and Research Assistant at the Brazilian Cochrane Center, São Paulo, Brazil.; III MD, MSc, PhD. Professor at Universidade Federal do Rio Grande do Norte (UFRN), Natal, Rio Grande do Norte, and Research Assistant at the Brazilian Cochrane Center, São Paulo, Brazil.

**Keywords:** Anti-inflammatory agents, Analgesia, Cyclooxygenase 2 inhibitors, Pain, Review [publication type], Antiinflamatórios, Analgesia, Inibidores de ciclooxigenase 2, Dor, Revisão

## Abstract

**CONTEXT AND OBJECTIVE:**

Lumiracoxib is an anti-inflammatory drug that has been used to treat acute dental pain, mainly in postsurgical settings, in which the greatest levels of pain and discomfort are experienced during the first 24 hours. This study aimed to assess the efficacy and safety of lumiracoxib for treating acute postsurgical dental pain.

**DESIGN AND SETTING:**

Systematic review developed at the Brazilian Cochrane Centre, Universidade Federal de São Paulo.

**METHODS:**

An electronic search was conducted in the PubMed, Cochrane Library, Lilacs (Literatura Latino-Americana e do Caribe em Ciências da Saúde), SciELO (Scientific Electronic Library Online) and Embase databases. A manual search was also performed. Only randomized controlled trials were included, and these were selected and assessed by two researchers with regard to the risk of bias.

**RESULTS:**

Three clinical trials with 921 participants were included. Lumiracoxib 400 mg produced onset of analgesia in a shorter time than shown by lumiracoxib 100 mg, celecoxib 200 mg and ibuprofen 400 mg. There was no difference between lumiracoxib 400 mg and rofecoxib 50 mg. In two studies, the mean time taken to attain onset of analgesia for the placebo was not estimated because the number of participants who reached onset was too small.

**CONCLUSION:**

There is evidence with a moderate risk of bias that recommends the use of lumiracoxib for acute postoperative dental pain. However, the adverse effects are not completely known. Given that lumiracoxib is currently available in only three countries, further studies are likely to be rare and discouraged.

## RESUMO

**CONTEXTO E OBJETIVO:**

O lumiracoxibe é um anti-inflamatório que tem sido utilizado no tratamento de dor dental aguda, principalmente no cenário pós-cirúrgico, no qual níveis mais elevados de dor e desconforto são sentidos durante as primeiras 24 horas. Este estudo teve por objetivo avaliar a eficácia e a segurança do lumiracoxibe no tratamento da dor dental aguda e pós-operatória.

**TIPO DE ESTUDO E LOCAL:**

Revisão sistemática desenvolvida no Centro Cochrane do Brasil, Universidade Federal de São Paulo.

**MÉTODOS:**

Foi realizada busca eletrônica nas bases de dados PubMed, Cochrane Library, Lilacs (Literatura Latino-Americana e do Caribe em Ciências da Saúde), SciELO (Scientific Electronic Library Online) e Embase. Também foi realizada busca manual. Apenas ensaios clínicos randomizados foram incluídos e foram selecionados e avaliados por dois pesquisadores quanto ao risco de viés.

**RESULTADOS:**

Foram incluídos três ensaios clínicos com 921 participantes. O lumiracoxibe 400 mg mostrou menor tempo de início de analgesia que o lumiracoxibe 100 mg, celecoxibe 200 mg e ibuprofeno de 400 mg. Não houve diferença entre lumiracoxibe 400 mg e rofecoxibe 50 mg. Em dois estudos o tempo médio de início de analgesia para o placebo não foi estimado, pois o número de participantes que a alcançou foi pequeno.

**CONCLUSÃO:**

Há evidências, com moderado risco de viés, que recomendam o uso de lumiracoxibe para a dor dental aguda e pós-operatória. No entanto, os efeitos adversos não são completamente conhecidos. Considerando que o lumiracoxibe está disponível em apenas três países, provavelmente a realização de pesquisas futuras será rara e desestimulada.

## INTRODUCTION

Pain is an unpleasant sensory and emotional experience associated with actual or potential tissue damage, or described in terms of such damage. Acute pain is elicited by substantial injury of body tissue and activation of nociceptive transducers at the site of local tissue damage. This type of pain is also seen after trauma, surgical interventions and some diseases.^1^ Also in dentistry, this pain may be associated with a pathological condition or a surgical intervention. Among the most common causes for acute toothache of pathological origin are acute pulp pain and acute periodontal pain. Acute pulp pain may come from dental exposure or from bacterial infection in the dental pulp. Periodontal acute pain is usually due to acute apical periodontitis resulting from trauma or extension of pulp inflammation inwards to apical tissues, or may be due to acute periodontal abscess or acute periodontitis, usually of bacterial origin.^2^ Among dental procedures, surgical removal of wisdom teeth and periodontal surgical procedures are the ones most expected to cause postoperative pain.^3^

Acute dental pain and postoperative pain are often treated with analgesics and non-steroidal anti-inflammatory drugs (NSAIDs) such as paracetamol, ibuprofen, rofecoxib and celecoxib.^4-6^ The efficacy of these drugs in reducing inflammation and pain is largely attributed to prevention of prostaglandin synthesis via non-specific inhibition of both cyclooxygenase-1 (COX-1) and cyclooxygenase-2 (COX-2).^5^

Lumiracoxib is a new selective inhibitor of COX-2 that was also developed for treating acute pain. COX-2 inhibitors have been developed to avoid COX-1 related gastrointestinal problems.^7^ Lumiracoxib is structurally distinct from other COX-2 selective inhibitors; it has demonstrated good oral bioavailability (74%), rapid absorption and a relatively short plasma half-life of 3-6 hours. In spite of the relatively short half-life, studies have shown that lumiracoxib administered once daily provides 24-hours analgesic efficacy.^5^ It has been postulated that the analgesic actions of NSAIDs result from inhibition of peripheral synthesis of prostaglandins, which are formed secondarily to tissue injury and inflammation by the enzyme cyclooxygenase.^8^ Patients who have undergone dental surgery report high levels of pain and discomfort during the first 24 hours.^5,6^ It is therefore essential that any analgesic agent used to treat postoperative pain should have rapid onset of action on the first day following dental surgery.

Lumiracoxib is currently available in three countries, and the permitted doses are restricted to short prescriptions. Therefore, this study assessed the effects of lumiracoxib in relieving acute postoperative dental pain in an attempt to provide dentists with a drug for which dosing and ideal action timing information are available, and also safety information for patients.

## OBJECTIVES

To develop a systematic review assessing the efficacy and safety of lumiracoxib for treating acute postoperative dental pain.

## METHODS

This systematic review was developed at the Brazilian Cochrane Center and within the Postgraduate Program on Internal and Therapeutic Medicine at Universidade Federal de São Paulo (Unifesp), after receiving approval from the local Ethics Committee. The review and meta-analysis were conducted in accordance with the Cochrane Collaboration Handbook.^8^

### Data sources and searches

The following electronic databases were searched: CENTRAL (Cochrane Central Register of Controlled Trials), PubMed (1966 to present), Embase (1980 to present), Lilacs (Literatura Latino-Americana e do Caribe em Ciências da Saúde, 1982 to present), SciELO (Scientific Electronic Library Online), the Cochrane Oral Health Group's Specialized Register and the Cochrane Pain, Palliative and Supportive Care Specialized Register. A general search strategy was used, with adaptations to the characteristics of each database. The search strategies used are presented in Table 1.

**Table 1:** Search strategies for electronic database

Database	Search strategy
Cochrane Library	1. lumiracoxib
	2. prexige
	3. OR/1-2
	4. Exp PAIN (MeSH term)
	5. pain$
	6. toothache$
	7. odontalgi$
	8. OR/4-7
	9. SURGERY, ORAL (MeSH term)
	10. Exp ORAL SURGICAL PROCEDURES (MeSH term)
	11. ("oral surgical" or "oral surgery" or (dental AND (surgery or surgical)))
	12. (apicectom$ or apicoectom$ or (osteotom$ AND (mandib$ or maxilla$)))
	13. ((tooth adj6 extract$) or "dental extract$" or (extract$ adj6 teeth))
	14. MOLAR, THIRD (MeSH term)
	15. ((third adj3 molar$) or third-molar$))
	16. "wisdom tooth" or "wisdom teeth"
	17. OR/14-16
	18. 9 or 10 or 11 or 12 or 13 or 17
	19. 3 AND 8 AND 18 (records retrieved = 9 records? test group 1)
	20. "COX 189" or COX-189
	21. CYCLOOXYGENASE 2 INHIBITORS (MeSH term)
	22. "COX-2 inhibitor$" or "cyclooxygenase 2 inhibitor$"
	23. OR/20-22
	24. (3 OR 23) AND 8 AND 18 (records retrieved = 73) (24 NOT 19 = 64 records test group 2)
	25. exp CYCLOOXYGENASE INHIBITORS (MeSH term)
	26. CYCLOOXYGENASE INHIBITORS (MeSH term)
	27. cyclooxygenase inhibitor$
	28. OR/26-27
	29. (3 OR 23 OR 28) AND 8 AND 18 (records retrieved = 105) (29 NOT 24 = 32 records test group 3)
Embase	1. lumiracoxib
	2. prexige
	3. OR/1-2
	4. Exp PAIN (MeSH term)
	5. pain$
	6. toothache$
	7. odontalgi$
	8. OR/4-7
	9. SURGERY, ORAL (MeSH term)
	10. Exp ORAL SURGICAL PROCEDURES (MeSH term)
	11. ("oral surgical" or "oral surgery" or (dental AND (surgery or surgical)))
	12. (apicectom$ or apicoectom$ or (osteotom$ AND (mandib$ or maxilla$)))
	13. ((tooth adj6 extract$) or "dental extract$" or (extract$ adj6 teeth))
	14. MOLAR, THIRD (MeSH term)
	15. ((third adj3 molar$) or third-molar$))
	16. "wisdom tooth" or "wisdom teeth"
	17. OR/14-16
	18. 9 or 10 or 11 or 12 or 13 or 17
	19. 3 AND 8 AND 18 (records retrieved = 9 records? test group 1)
	20. "COX 189" or COX-189
	21. CYCLOOXYGENASE 2 INHIBITORS (MeSH term)
	22. "COX-2 inhibitor$" or "cyclooxygenase 2 inhibitor$"
	23. OR/20-22
	24. (3 OR 23) AND 8 AND 18 (records retrieved = 73) (24 NOT 19 = 64 records test group 2)
	25. exp CYCLOOXYGENASE INHIBITORS (MeSH term)
	26. CYCLOOXYGENASE INHIBITORS (MeSH term)
	27. cyclooxygenase inhibitor$
	28. OR/26-27
	29. (3 OR 23 OR 28) AND 8 AND 18 (records retrieved = 105) (29 NOT 24 = 32 records test group 3)
	AND
	1. Randomized controlled trial/
	2. Controlled study/
	3. Randomization/
	4. Double blind procedure/
	5. Single blind procedure/
	6. Clinical trial/
	7. (clinical adj5 trial$).ti,ab,hw
	8. ((doubl$ or singl$ or tripl$ or trebl$) adj5 (blind$ or mask$)).ti,ab,hw
	9. Placebo/
	10. Placebo$.ti,ab,hw
	11. Random$.ti,ab,hw
	12. Methodology.sh
	13. latin square.ti,ab,hw
	14. crossover.ti,ab,hw
	15. cross-over.ti,ab,hw
	16. Crossover Procedure/
	17. Drug comparison/
	18. Comparative study/
	19. (comparative adj5 trial$).ti,ab,hw
	20. (control$ or prospectiv$ or volunteer$).ti,ab,hw
	21. exp "Evaluation and Follow Up"/
	22. Prospective study/
	23. or/1-22 24. animal/ not (human/ and animal/)25.23 not 24
	24. animal/ not (human/ and animal/)
	25. 23 not 24
Lilacs (Literatura Latino-Americana e do Caribe em Ciência da Saúde)	(prexige) OR (LUMIRACOXIB) OR (LUMIRACOXIB COX 189) OR (COX-189) OR (lumiracoxib) OR (COX-2 inhibitor) OR (Organic Chemicals) OR ("Cyclooxygenase 2 Inhibitors)
	(Toothache) OR (Toothaches) OR (Odontalgia) OR (Odontalgias) OR (acute and post-operation dental pain) OR (acute dental pain)
PubMed	("prexige "[Substance Name]) OR (LUMIRACOXIB) OR (LUMIRACOXIB COX 189) OR (COX-189) OR (lumiracoxib) OR (COX-2 inhibitor) OR ("Organic Chemicals"[Mesh]) OR ("Cyclooxygenase 2 Inhibitors "[Pharmacological Action]) or (Cyclooxygenase Inhibitors)
	AND
	("Toothache"[Mesh]) OR (Toothaches) OR (Odontalgia) OR (Odontalgias) OR (acute and post-operation dental pain) OR (acute dental pain) or (dental pain postoperative)
	AND
	randomized controlled trial [Publication Type] OR controlled clinical trial [Publication Type] OR randomized controlled trials [MeSH Terms] OR random allocation [MeSH Terms] OR double blind method [MeSH Terms] OR single blind method [MeSH Terms] OR clinical trial [Publication Type] OR clinical trials [MeSH Terms] OR (clinical* [Text Word] AND trial* [Text Word]) OR single* [Text Word] OR double* [Text Word] OR treble* [Text Word] OR triple* [Text Word] OR placebos [MeSH Terms] OR placebo* [Text Word] OR random* [Text Word] OR research design [MeSH Terms] OR comparative study [MeSH Terms] OR evaluation studies [MeSH Terms] OR follow-up studies [MeSH Terms] OR prospective studies [MeSH Terms] OR control* [Text Word] OR prospectiv* [Text Word] OR volunteer* [Text Word]
SciELO	(prexige) OR (LUMIRACOXIB) OR (LUMIRACOXIB COX 189) OR (COX-189) OR (lumiracoxib) OR (COX-2 inhibitor) OR (Organic Chemicals) OR ("Cyclooxygenase 2 Inhibitors)
	AND
	(Toothache) OR (Toothaches) OR (Odontalgia) OR (Odontalgias) OR (acute and post-operation dental pain) OR (acute dental pain)
Cochrane Library	1. lumiracoxib
	2. prexige
	3. OR/1-2
	4. Exp PAIN (MeSH term)
	5. pain$
	6. toothache$
	7. odontalgi$
	8. OR/4-7
	9. SURGERY, ORAL (MeSH term)
	10. Exp ORAL SURGICAL PROCEDURES (MeSH term)
	11. ("oral surgical" or "oral surgery" or (dental AND (surgery or surgical)))
	12. (apicectom$ or apicoectom$ or (osteotom$ AND (mandib$ or maxilla$)))
	13. ((tooth adj6 extract$) or "dental extract$" or (extract$ adj6 teeth))
	14. MOLAR, THIRD (MeSH term)
	15. ((third adj3 molar$) or third-molar$))
	16. "wisdom tooth" or "wisdom teeth"
	17. OR/14-16
	18. 9 or 10 or 11 or 12 or 13 or 17
	19. 3 AND 8 AND 18 (records retrieved = 9 records? test group 1)
	20. "COX 189" or COX-189
	21. CYCLOOXYGENASE 2 INHIBITORS (MeSH term)
	22. "COX-2 inhibitor$" or "cyclooxygenase 2 inhibitor$"
	23. OR/20-22
	24. (3 OR 23) AND 8 AND 18 (records retrieved = 73) (24 NOT 19 = 64 records test group 2)
	25. exp CYCLOOXYGENASE INHIBITORS (MeSH term)
	26. CYCLOOXYGENASE INHIBITORS (MeSH-0 term)
	27. cyclooxygenase inhibitor$
	28. OR/26-27 29. (3 OR 23 OR 28) AND 8 AND 18 (records retrieved = 105) (29 NOT 24 = 32 records test group 3)
Embase	1. lumiracoxib
	2. prexige
	3. OR/1-2
	4. Exp PAIN (MeSH term)
	5. pain$
	6. toothache$
	7. odontalgi$
	8. OR/4-7
	9. SURGERY, ORAL (MeSH term)
	10. Exp ORAL SURGICAL PROCEDURES (MeSH term)
	11. ("oral surgical" or "oral surgery" or (dental AND (surgery or surgical)))
	12. (apicectom$ or apicoectom$ or (osteotom$ AND (mandib$ or maxilla$)))
	13. ((tooth adj6 extract$) or "dental extract$" or (extract$ adj6 teeth))
	14. MOLAR, THIRD (MeSH term)
	15. ((third adj3 molar$) or third-molar$))
	16. "wisdom tooth" or "wisdom teeth"
	17. OR/14-16 18. 9 or 10 or 11 or 12 or 13 or 17 19. 3 AND 8 AND
	18 (records retrieved = 9 records? test group 1)
	19. 3 AND 8 AND 18 (records retrieved = 9 records? test group 1)
	20. "COX 189" or COX-189
	21. CYCLOOXYGENASE 2 INHIBITORS (MeSH term)
	22. "COX-2 inhibitor$" or "cyclooxygenase 2 inhibitor$"
	23. OR/20-22
	24. (3 OR 23) AND 8 AND 18 (records retrieved = 73) (24 NOT 19 = 64 records test group 2)
	25. exp CYCLOOXYGENASE INHIBITORS (MeSH term)
	26. CYCLOOXYGENASE INHIBITORS (MeSH term)
	27. cyclooxygenase inhibitor$
	28. OR/26-27
	29. (3 OR 23 OR 28) AND 8 AND 18 (records retrieved = 105) (29 NOT 24 = 32 records test group 3)
	AND
	1. Randomized controlled trial/
	2. Controlled study/
	3. Randomization/
	4. Double blind procedure/
	5. Single blind procedure/
	6. Clinical trial/
	7. (clinical adj5 trial$).ti,ab,hw
	8. ((doubl$ or singl$ or tripl$ or trebl$) adj5 (blind$ or mask$)).ti,ab,hw
	9. Placebo/
	10. Placebo$.ti,ab,hw
	11. Random$.ti,ab,hw
	12. Methodology.sh
	13. latin square.ti,ab,hw
	14. crossover.ti,ab,hw
	15. cross-over.ti,ab,hw
	16. Crossover Procedure/
	17. Drug comparison/
	18. Comparative study/
	19. (comparative adj5 trial$).ti,ab,hw
	20. (control$ or prospectiv$ or volunteer$).ti,ab,hw
	21. exp "Evaluation and Follow Up"/
	22. Prospective study/
	23. or/1-22
	24. animal/ not (human/ and animal/)
	25. 23 not 24
Lilacs (Literatura Latino-Americana e do Caribe em Ciência da Saúde)	(prexige) OR (LUMIRACOXIB) OR (LUMIRACOXIB COX 189) OR (COX-189) OR (lumiracoxib) OR (COX-2 inhibitor) OR (Organic Chemicals) OR (Cyclooxygenase 2 Inhibitors)
	(Toothache) OR (Toothaches) OR (Odontalgia) OR (Odontalgias) OR (acute and post-operation dental pain) OR (acute dental pain)
PubMed	("prexige "[Substance Name]) OR (LUMIRACOXIB) OR (LUMIRACOXIB COX 189) OR (COX-189) OR (lumiracoxib) OR (COX-2 inhibitor) OR ("Organic Chemicals"[Mesh]) OR ("Cyclooxygenase 2 Inhibitors "[Pharmacological Action]) or (Cyclooxygenase Inhibitors)
	AND
	("Toothache"[Mesh]) OR (Toothaches) OR (Odontalgia) OR (Odontalgias) OR (acute and post-operation dental pain) OR (acute dental pain) or (dental pain postoperative)
	AND
	randomized controlled trial [Publication Type] OR controlled clinical trial [Publication Type] OR randomized controlled trials [MeSH Terms] OR random allocation [MeSH Terms] OR double blind method [MeSH Terms] OR single blind method [MeSH Terms] OR clinical trial [Publication Type] OR clinical trials [MeSH Terms] OR (clinical* [Text Word] AND trial* [Text Word]) OR single* [Text Word] OR double* [Text Word] OR treble* [Text Word] OR triple* [Text Word] OR placebos [MeSH Terms] OR placebo* [Text Word] OR random* [Text Word] OR research design [MeSH Terms] OR comparative study [MeSH Terms] OR evaluation studies [MeSH Terms] OR follow-up studies [MeSH Terms] OR prospective studies [MeSH Terms] OR control* [Text Word] OR prospectiv* [Text Word] OR volunteer* [Text Word]
SciELO	(prexige) OR (LUMIRACOXIB) OR (LUMIRACOXIB COX 189) OR (COX-189) OR (lumiracoxib) OR (COX-2 inhibitor) OR (Organic Chemicals) OR (Cyclooxygenase 2 Inhibitors)
	AND
	(Toothache) OR (Toothaches) OR (Odontalgia) OR (Odontalgias) OR (acute and post-operation dental pain) OR (acute dental pain)

For the manual search, thesis databases, references lists of the relevant studies and clinical trials register databases were taken into account. Specialists in the field, authors of the trials included and pharmaceutical companies were contacted in order to try to obtain unpublished or missing data.

There was no language restriction.

Randomized clinical trials were included. In these trials, the participants were adults suffering from acute postoperative pain who received lumiracoxib (either separately or in association), regardless of the dosage, and the use of this drug was compared with other interventions, such as anti-inflammatory drugs, analgesics or placebo. The trials included needed to evaluate improvement of pain as an outcome, regardless of the tool used to assess this.

### Study selection

Study selection was performed independently by two reviewers (RS and RR), in order to identify and select studies that met the inclusion criteria for this review. Agreement was reached by consensus after analysis of the complete text of the study, and after contact with study authors for further information, when needed.

### Data extraction and risk-of-bias assessment

Two authors (RS and RR) independently extracted the data and used a standard form to organize the following information regarding each study included: title, publication data, authors, number and characteristics of the participants, interventions, outcomes and potential conflicts of interests among the authors involved in the trial.

The risks of bias of the trials included were measured independently by two reviewers using the risk-of-bias table developed by the Cochrane Collaboration, which is available in the Cochrane Handbook.^9^ Six domains were assessed: sequence generation, allocation concealment, blinding of participants, personnel and outcome assessors, incomplete outcome data, freedom from selective reporting and other sources of bias. These domains were classified as "Yes" (i.e. low risk of bias), "Unclear" (uncertain risk of bias) or "No" (i.e. high risk of bias). The overall classification of each study was based on the three main domains as follows: sequence generation, allocation concealment and blinding, as stated in the Handbook for Systematic Reviews.^9^

The evaluations were compared and any inconsistencies between the review authors in interpreting the inclusion criteria and their significance for the trials selected were discussed and resolved. The study authors were contacted in cases of missing data or uncertainty over any data.

### Data analysis

The Review Manager software 5.0 (Cochrane Collaboration) was used to produce the graphs. For dichotomous outcomes, the intervention estimates were expressed as relative risks together with 95% confidence intervals. For continuous outcomes, mean differences and 95% confidence intervals were used to summarize the data for each trial.

Since it was not possible to produce any meta-analysis, considering that the three studies included were heterogeneous in relation to comparison groups, outcomes assessed or tools applied, heterogeneity and sensitivity analyses were not carried out.

## RESULTS

Through the search strategy, 279 articles were found. From these, based on the inclusion criteria, only four were selected. There was one case of duplicated publication, and therefore one clinical trial was excluded (study by Schnitzer).^10^

**Figure 1:**
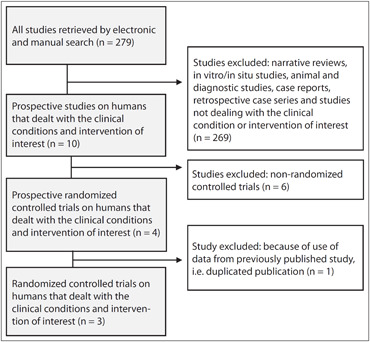
Flow chart of studies, from databases to inclusion in the systematic review.

The first study included, with 202 patients, presented four arms and compared lumiracoxib 400 mg or 100 mg with ibuprofen 400 mg and placebo.^6^ The second one compared lumiracoxib 400 mg with rofecoxib 50 mg, celecoxib 200 mg and placebo, and assessed 355 patients.^5^ The third compared lumiracoxib 400 mg with celecoxib 400 mg and placebo, and assessed 364 patients.^4^ These studies were all carried out in the United States with financial support from the pharmaceutical industry. The overall flow chart of the studies is presented in Figure 1.

The efficacy outcomes that it was initially proposed to assess through these trials were: time taken to onset of analgesia, overall evaluation score, relief of pain on visual analogue scale (VAS), four-point category scale pain, summed (time-weighted) pain intensity difference (SPID), pain relief (PR) and pain relief intensity difference (PRID).

Despite this considerable number of outcomes, many of them were presented incompletely, for instance: a) upper and low limits were not available to calculate the standard deviation (SD); or b) the results were presented in graphic form with no raw data available. The authors were contacted sometimes, with the aim of resolving these inconsistencies, but without reply.

The studies also evaluated adverse effects, vital signs and clinical laboratory tests. The adverse effects are described later on in this paper. The other matters that these studies assessed were not within the aims of the present review, which were to evaluate clinical outcomes relating to efficacy and safety.

**Figure 2:**
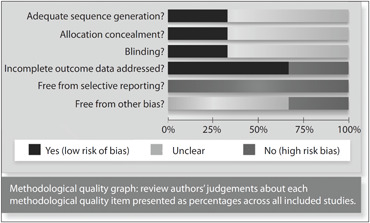
Risk of bias in included studies.

**Figure 3:**
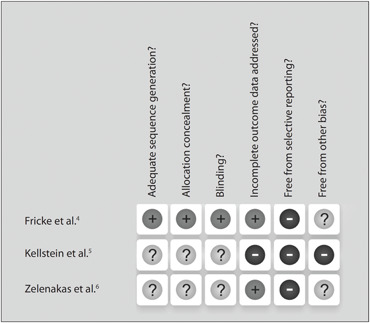
Methodological quality summary: review authors’ judgments about each methodological quality item for each study included.

As stated in the methods section, the risk of bias of each included study was assessed through the risk-of-bias table from the Cochrane Collaboration.^9^ The studies were classified as presenting moderate risk of bias, since all the three key domains (sequence generation, allocation concealment and blinding) were considered "unclear", as presented in Figures 2 and 3.

### 1. Sequence generation

Two studies that were included were considered to have a moderate risk of bias.^5,6^ Although they were reported to be randomized, the method used to generate the allocation sequence was not presented. There was an attempt to contact their authors, but no reply was obtained from them. One study (Fricke et al.) was considered to present a low risk of bias.^4^

### 2. Allocation concealment

In two of the studies included, the method used by the authors to conceal the allocation sequence was not described in sufficient detail to be able to determine whether the interventions had been planned in advance or during registration.^5,6^ One study reported the allocation concealment method.^4^

### 3. Blinding of participants, personnel and outcome assessors

The three studies were described as double-blind. However, in the trial by Zelenakas et al., there was insufficient information to allow judgment about the adequacy of the blinding and also, the author did not reply to electronic mail enquiries asking for related information.^6^ The trial by Kellstein et al. was described as a double-dummy design in which each patient received two tablets (either lumiracoxib or matching placebo) and one capsule (rofecoxib, celecoxib or matching placebo).^5^ Since both of the active comparators were over-encapsulated in order to maintain blinding, dissolution tests were performed to confirm that there was no clinically meaningful effect from encapsulation on comparator dissolution. In the study by Fricke et al., the method used for blinding was described.^4^

### 4. Incomplete outcome data

In the study by Kellstein et al., an intention-to-treat (ITT) analysis was conducted, and no dropout was reported.^5^ If required, patients were allowed to take rescue medication consisting of hydrocodone bitartrate/paracetamol (5 mg/500 mg) or paracetamol 1000 mg at any time during the 24-hour post-dose period. The time of rescue use was recorded, and no further efficacy evaluations were performed afterwards. However, the number of patients who required rescue medication was not reported. In the study by Zelenakas et al., an ITT analysis was also performed, and there were 145 dropouts. The reasons for medication and early discontinuation were presented.^6^

### 5. Selective reporting

In all three studies, the outcomes were reported incompletely. The study by Zelenakas et al. did not inform all the data, including the following outcome data: PID, PRID and SPID.^6^ In the study by Kellstein et al, the results relating to PI and PRID (which had been proposed in the methods section) were not reported.^5^ In the study by Fricke et al., the median time taken to attain the onset of analgesia was not reported for either celecoxib or the placebo.^4^

### 6. Other sources of bias

In all the studies, there was insufficient information to assess whether any other important risk of bias existed or not.

### Outcome measurements

#### Time taken to attain the onset of analgesia

This was the only efficacy outcome measurement that could be fully assessed. Lumiracoxib 400 mg showed a shorter time taken to attain the onset of analgesia than shown by lumiracoxib 100 mg (mean difference, MD = −15.00; 95% confidence interval, CI: −100.05 to 70.05; P = 0.73). The time to onset of analgesia also favored lumiracoxib at a dose of 400 mg, compared with celecoxib 200 mg (MD = −11.34; 95% CI: −16.83 to −5.85; P < 0.0001) and with ibuprofen 400 mg (MD = −4.10; 95% CI: −19.59 to 11.39; P = 0.60). A statistically non-significant MD was observed between lumiracoxib 400 mg and rofecoxib 50 mg (MD = −0.19; 95% CI: −5.82 to 5.44; P = 0.95). In all of the studies, the median time to onset for the placebo could not be estimated because the number of participants who achieved onset was too small. In Fricke's study, the time taken to attain the onset of analgesia was reported for lumiracoxib 400 mg (38 minutes), but not for celecoxib 400 mg. The median difference in time taken to attain the onset of analgesia using lumiracoxib could not be calculated because the authors did not report the CI to enable calculation of a standard deviation (SD).

**Figure 4:**
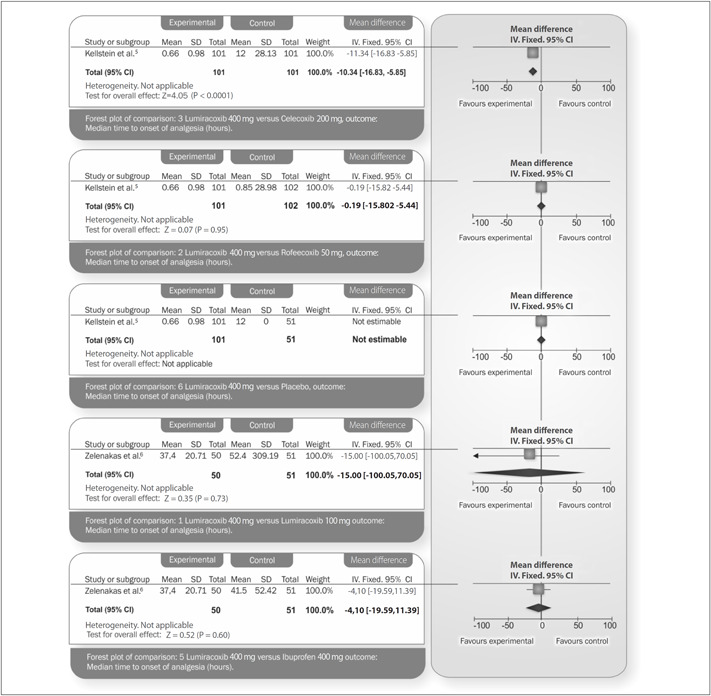
Median time taken to attain the onset of analgesia.

The graph presented in Figure 4 shows the median time taken for onset of analgesia (primary efficacy outcome).

#### Use of rescue medication

One study reported the number of patients who had required rescue medication within 12 hours.^5^ The number of patients who had required rescue medication was lower in all the active treatment groups than in the placebo group (92.0%) (risk ratio, RR = 0.25; 95% CI: 0.08 to 0.84; P = 0.03). Similar proportions of patients in the lumiracoxib 100 mg group (74.5%) and ibuprofen 400 mg group (72.5%) required rescue medication (RR = 1.03; 95% CI: 0.81 to 1.3; P = 0.82). This proportion was numerically lower in the lumiracoxib 400 mg group (44.0%) (RR = 0.59; 95% CI: 0.42 to 0.84; P = 0.003).

The time elapsed before rescue medication was required was significantly longer in all of the active treatment groups than in the placebo group (median time = 12 hours; P < 0.001), while the time elapsed before rescue medication was required for lumiracoxib 400 mg (median time = 12 hours) was significantly longer than for lumiracoxib 100 mg (median time = 7 hours; P < 0.01) and ibuprofen 400 mg (median time = 8 hours; P < 0.01), as presented in Figure 4.

#### Adverse events

All the clinical trials presented information on the number of patients with one or more adverse events. Twenty-four hours after intake, Kellstein et al. reported an adverse event rate of 21/101 patients with lumiracoxib 400 mg and 9/51 with placebo (RR = 1.18; 95% CI: 0.58 to 2.38; P = 0.2).^5^ Twelve hours after intake, Zelenakas et al. reported a rate of 1/50 (2%) with lumiracoxib 400 mg and 10/50 with placebo (RR = 0.1; 95% CI: 0.01 to 0.75; P = 0.02).^6^ Twenty-four hours after intake, Fricke et al. reported a rate of 18/156 with lumiracoxib 400 mg and 9/52 with placebo (RR = 0.67; 95% CI: 0.32 to 1.39; P = 0.8).^4^

There was only one withdrawal in one study.^6^ This patient had been allocated to an ibuprofen group and presented postoperative bleeding at the suture site, but no association with the drug was reported. There was only one serious adverse event, in a patient who had received placebo, who presented deep vein thrombosis (DVT).^6^

The adverse events were generally described as mild to moderate in severity. Most of them were probably related to the patients’ postoperative status. It is noteworthy that none of the studies included reported any cases of kidney failure. More details about other adverse events are shown in Table 2.

**Table 2:** Adverse events

Adverse Events	Lumiracoxib 400 mg % (n/N)	Lumiracoxib 100 mg % (n/N)	Rofecoxib 50 mg % (n/N)	Celecoxib 200 mg % (n/N)	Celecoxib 400 mg % (n/N)	Ibuprofen 400 mg % (n/N)	Placebo % (n/N)
Deep vein thrombosis	0 (0/50)	0 (0/51)	_	_	_	0 (0/51)	2 (1/50)
GI signs and symptoms*	9 (27/307)	4 (4/51)	12 (12/102)	11 (20/101)	11(17/156)	8 (5/51)	14 (21/153)
Dizziness (excluding vertigo)	4 (10/257)	_	1 (1/102)	4 (4/101)	2 (3/156)	_	4 (4/103)
Vomiting	7 (17/257)	_	5.9 (6/102)	5.9 (6/101)	3 (5/156)	_	10 (10/103)
Headache	4 (9/257)	_	0 (0/102)	4 (4/101)	6 (9/156)	_	5 (5/103)
Increased sweating	1 (1/101)	_	0 (0/102)	2 (2/101)	_	_	2 (1/51)
Abdominal pain	2 (2/101)	_	1 (1/102)	0 (0/101)	_	_	0 (0/51)
Dyspnea	0 (0/101)	_	0 (0/102)	0 (0/101)	_	_	2 (1/51)
Feeling jittery	1 (1/101)	_	0 (0/102)	0 (0/101)	_	_	2 (1/51)
Syncope	0 (0/156)	_	_	_	1 (1/156)	_	0 (0/0)
Pyrexia	1 (1/156)	_	_	_	0 (0/156)	_	0 (0/0)
Total number of patients with any adverse event	13 (41/307)	14 (7/100)	12 (12/102)	20 (20/101)	11 (17/156)	10 (5/51)	18 (28/153)

n = number of patients who presented the event; N = number of patients; GI = gastrointestinal;

* such as nausea, emesis and diarrhea.

## DISCUSSION

This study, based on analysis of the clinical trials that had been included in it, showed that lumiracoxib 400 mg was more effective when used for acute dental pain and postoperative pain than were placebo, lumiracoxib 100 mg, celecoxib 200 mg or ibuprofen 400 mg for the following outcomes: time taken to attain the onset of analgesia and time elapsed before rescue medication was required. There was no difference in relation to rofecoxib 50 mg. With regard to adverse events, this review included short-term trials, which only allowed assessment of acute effects. Moreover, the limited sample size was unhelpful for this type of analysis.

To our knowledge, this is the only systematic review assessing the efficacy and safety of lumiracoxib in relation to this issue. This review protocol was started at a time when lumiracoxib was available in several countries for many inflammatory or painful symptoms. However, some months later, the market scenario for lumiracoxib changed such that this medication only remained in current use in three countries: Mexico, Ecuador and the Dominican Republic. Moreover, it was withdrawn from several countries such as Brazil, Canada, the United Kingdom and Australia because of important adverse effects that had been correlated with its use.^11-13^

With regard to the implications for research on single-dose drugs for treating pain, clinical trials with methodology that is appropriate for consistent evaluation are recommended.

By using a sensitive search strategy, 279 articles were found. Among them, four articles met the inclusion criteria. It should be noted that one of the four articles selected was excluded (study by Schnitzer et al.)^10^ because it used data from a study previously published (Zelenakas et al.).^6^

The results from clinical trials may, in practice, be limited. This can be seen from the study by Zelenakas et al., in which 57 patients (28.2%) completed the study out of 202 patients who had been randomized.^6^ Even though a patient loss of 71.8% was demonstrated, this study was included in the present review.

Regarding the results from continuous variables, the overall effect measured only by comparing the means after the treatment may not be representative of the sample. This might be considered to be a limitation of the studies included in this review. The ideal would be to compare the groups as either the percentage of patients who showed improvements in values (after dichotomizing the continuous variable) or the mean difference achieved (improvement) by each intervention group after the treatment. Through these statistical analyses, it would be possible to obtain the relative risk and the number need to treat, which would certainly be more useful in clinical practice.

The lack of data limits some of the results from this review. The study by Fricke et al. did not provide SD values, or even the CI values (which could be used to obtain SD values indirectly) for the ‘median time taken to attain the onset of analgesia’ outcome.^4^ Hence, it was not possible to pool this study in the graph presented in Figure 4.

These studies presented moderate risk of bias, according to the Cochrane Collaboration's tool for assessing the risk of bias, which relates to moderate risk of uncertainty among the results available. Indeed, many of the efficacy outcomes proposed in the methods section of each study were not adequately presented in the results section, which led to notable reporting bias. Despite this, the present review can be considered to be the best current evidence so far developed to try to answer the questions: Does lumiracoxib present efficacy with regard to acute postoperative dental pain? Is it safe for this setting?

With regard to implications for further research on adverse event profiles, there is a lack of long-term studies, given that the duration of acute treatment is short or limited. Lumiracoxib use is time-limited or restricted, since this medication today is only marketed in three countries. Hence, it will be difficult to find further and better evidence regarding efficacy and safety. Moreover, it can be highlighted that the incomplete data presentation was an important barrier preventing strong statistical findings. Such barriers may be a considerable source of bias, and this needs to be avoided in further studies.

With regard to implications for practice, considering the results from this review, there is some evidence, with a moderate risk of bias, that lumiracoxib can be recommended for treating acute postoperative dental pain. Dental surgeons need a drug that has a rapid analgesic effect, in order to rehabilitate and restore their patients’ quality of life quickly and convincingly. This medication should be prescribed for a short period of time and only be used to treat acute postsurgical pain. However, the adverse effects are not fully known and dental professionals need to have enough evidence to choose the best drug for treating their patients and, especially, not to jeopardize their safety.

## CONCLUSIONS

There is evidence, with a moderate risk of bias, that lumiracoxib can be recommended for treating acute postoperative dental pain. However, the adverse effects are not fully known. Given that this drug is currently available in only three countries, further research is likely to be rare and discouraged.
